# Distinct spatiotemporal immunometabolic remodeling following acute traumatic brain injury

**DOI:** 10.64898/2026.07.28.740519

**Published:** 2026-07-29

**Authors:** Zohreh Erfani, Erin H. Seeley, Erik J. Plautz, Brenda L. Bartnik-Olson, Jae Mo Park

## Abstract

Traumatic brain injury induces profound metabolic reprogramming across neurons, astrocytes, and microglia, yet the spatiotemporal organization of these metabolic responses remains poorly understood. Because pyruvate is uniquely positioned in cerebral metabolism by connecting glycolysis, lactate metabolism, and the tricarboxylic acid cycle, we combined matrix-assisted laser desorption/ionization (MALDI) mass spectrometry imaging with *in vivo* administration of isotopically labeled pyruvate and immunohistochemistry to characterize cell-type-wise metabolic remodeling in a controlled cortical impact rat model during the acute and subacute phases of injury. TBI induced distinct spatiotemporal immunometabolic remodeling across neuroglial compartments. Microglia- and macrophage-enriched regions exhibited selective accumulation of citrate, succinate, and itaconate, consistent with inflammatory metabolic rewiring. In contrast, astrocyte enriched regions showed increased glutamine and malate abundance, indicative of altered neuron-astrocyte metabolic coupling such as remodeling of the glutamate-glutamine cycle and enhanced anaplerotic metabolism. These metabolic signatures evolved with distinct regional and temporal distributions, identifying compartmentalized metabolic responses. Notably, labeled isotopologues of selected metabolites, including glutamate and citrate derived from administered pyruvate, changed before the corresponding metabolite pools. Together, these findings describe the spatiotemporal landscape of immunometabolic remodeling following acute TBI, uncover metabolically distinct microglial/macrophage and astrocytic responses during secondary brain injury, and identify candidate metabolic pathways for therapeutic intervention and metabolic imaging.

## Full Text Availability

The license terms selected by the author(s) for this preprint version do not permit archiving in PMC. The full text is available from the preprint server.

